# CART-ANOVA-Based Transfer Learning Approach for Seven Distinct Tumor Classification Schemes with Generalization Capability

**DOI:** 10.3390/diagnostics15030378

**Published:** 2025-02-05

**Authors:** Shiraz Afzal, Muhammad Rauf, Shahzad Ashraf, Shahrin Bin Md Ayob, Zeeshan Ahmad Arfeen

**Affiliations:** 1 Department of Electronic Engineering, Dawood University of Engineering and Technology, Karachi 74800, Pakistan; muhammad.rauf@duet.edu.pk; 2 Department of Computer Science, DHA Suffa University, Karachi 75500, Pakistan; 3 Faculty of Electrical Engineering, Universiti Teknologi Malaysia, Johor Bahru 81310, Malaysia; 4 Department of Electrical Engineering, The Islamia University of Bahawalpur (IUB), Bahawalpur 63100, Pakistan

**Keywords:** brain tumor, knowledge transfer learning, Cart-ANOVA, hyperparameter optimization, MRI Image, ResNet18, brain tumor classification

## Abstract

**Background/Objectives:** Deep transfer learning, leveraging convolutional neural networks (CNNs), has become a pivotal tool for brain tumor detection. However, key challenges include optimizing hyperparameter selection and enhancing the generalization capabilities of models. This study introduces a novel CART-ANOVA (Cartesian-ANOVA) hyperparameter tuning framework, which differs from traditional optimization methods by systematically integrating statistical significance testing (ANOVA) with the Cartesian product of hyperparameter values. This approach ensures robust and precise parameter tuning by evaluating the interaction effects between hyperparameters, such as batch size and learning rate, rather than relying solely on grid or random search. Additionally, it implements seven distinct classification schemes for brain tumors, aimed at improving diagnostic accuracy and robustness. **Methods:** The proposed framework employs a ResNet18-based knowledge transfer learning (KTL) model trained on a primary dataset, with 20% allocated for testing. Hyperparameters were optimized using CART-ANOVA analysis, and statistical validation ensured robust parameter selection. The model’s generalization and robustness were evaluated on an independent second dataset. Performance metrics, including precision, accuracy, sensitivity, and F1 score, were compared against other pre-trained CNN models. **Results:** The framework achieved exceptional testing accuracy of 99.65% for four-class classification and 98.05% for seven-class classification on the source 1 dataset. It also maintained high generalization capabilities, achieving accuracies of 98.77% and 96.77% on the source 2 datasets for the same tasks. The incorporation of seven distinct classification schemes further enhanced variability and diagnostic capability, surpassing the performance of other pre-trained models. **Conclusions:** The CART-ANOVA hyperparameter tuning framework, combined with a ResNet18-based KTL approach, significantly improves brain tumor classification accuracy, robustness, and generalization. These advancements demonstrate strong potential for enhancing diagnostic precision and informing effective treatment strategies, contributing to advancements in medical imaging and AI-driven healthcare solutions.

## 1. Introduction

**Figure 1:**
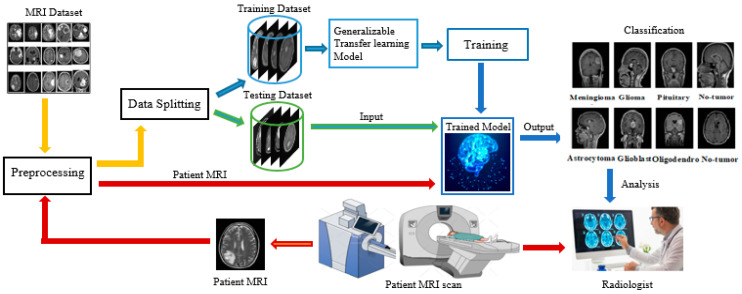
Typical methodology for “image analysis using computer vision”.

The human brain, one of the most complex and vital organs, controls nearly all cognitive and physiological functions. Any disruption to its normal functioning, such as from a neoplasm or tumor caused by abnormal cell growth, can have severe consequences, potentially impacting the entire body [1]. Brain tumors are acknowledged as one of the most common causes of mortality globally, with an estimated 612,000 cases reported specifically in Western populations [2]. Statistical analysis from the National Brain Tumor Foundation indicates that mortality associated with brain tumors has risen over the past three decades, emphasizing the importance of accurate and timely diagnosis to ensure effective treatment and improve patient outcomes. In recent years, there has been a growing emphasis on non-invasive diagnostic approaches for brain neoplasms, motivated by considerations of both efficacy and safety. Generally, brain tumors have been categorized by radiologists into two broad categories: (i) separating normal from abnormal images with the help of magnetic resonance and (ii) determining the types and stages of abnormal images. However, the wide variability in the intensity, size, and shape of a tumor [3] presents significant challenges in manual classification, as brain tumors often appear similar in MR images. Moreover, the manual procedure is highly dependent upon the skill of a radiologist, which could lead to human error and inconsistency in the analysis. Computer-aided automated (CAD) systems offer several advantages; they not only store a large amount of image data but also support prognosis, diagnosis, and pre-surgical and post-surgical decision-making, which is quite challenging using manual analysis of an MRI [4]. The CAD process begins with image preprocessing, where various filtering techniques are applied to enhance the image, followed by resizing the image according to the requirements of the specific model [3]. The patient’s MRI data are then analyzed by a trained model that has been previously trained on a large dataset, enabling the model to accurately predict the patient’s MRI results. The annotations predicted by the CAD system are then further reviewed by a radiologist to ensure final validation, as shown in Figure 1. These advancements, powered by ML, ANNs, and DL, highlight AI’s transformative impact on modern medicine, emphasizing its role in accurate, non-invasive diagnostics and improved patient care [5].

Deep learning models have gained much impetus mainly because of superior performance in tasks like image classification without a segmentation step. Deep learning, including CNNs, has outperformed the older traditional machine learning approach across most domains, like the detection of fetal QRS signals [6], and disease risk in the human genome identified through fuzzy deep learning networks [7,8]. Brain pathology has recently witnessed increasing demands for more accurate computational predictions of classified objects, which have turned towards deep learning techniques for tasks such as brain tumor MRI image classification and precise detection [9]. Deep learning’s success in these fields can be linked to advances in computer technology, particularly through the development of increasingly sophisticated learning algorithms, powerful GPUs, and large datasets [10,11,12]. Deep learning models, however, often lack the capacity for reuse or effective generalization across diverse tasks, especially when trained on data specific to a single classification task [13]. Therefore, these models often struggle with generalization across diverse tasks and datasets. Knowledge transfer learning (KTL), thus, has emerged as one of the robust techniques for handling the scarcity of data in several classification tasks [14,15]. Knowledge transfer learning (KTL) has emerged as a promising approach to overcome this limitation by fine-tuning pre-trained models on task-specific datasets, thereby improving efficiency and accuracy even under data-scarce conditions.

With the demonstrated effectiveness of KTL, it has emerged as a powerful tool for improving brain tumor detection and particularly for common tumor classifications such as “no tumor”, “pituitary”, “meningioma”, and “glioma”, due to the widespread availability of datasets. However, a significant gap remains in the confident classification of glioma subtypes named astrocytoma, glioblastoma, and oligodendroglioma. Precise categorization at this level is essential for accurate analysis and diagnosis, which directly influences treatment planning and outcomes. Existing research, addressing binary or limited tumor classification [16,17,18], has largely overlooked the further classification of gliomas. The challenge lies in the overlapping attributes of these subtypes, potentially leading to misdiagnosis and less effective treatment regimens. In this regard, the presented work fills this gap by applying transfer learning on nine different pre-trained CNN models to improve classification efficacy in brain tumor analysis focusing on seven categories: no tumor, meningioma, pituitary, glioma, astrocytoma, glioblastoma, and oligodendroglioma.

**Figure 2:**
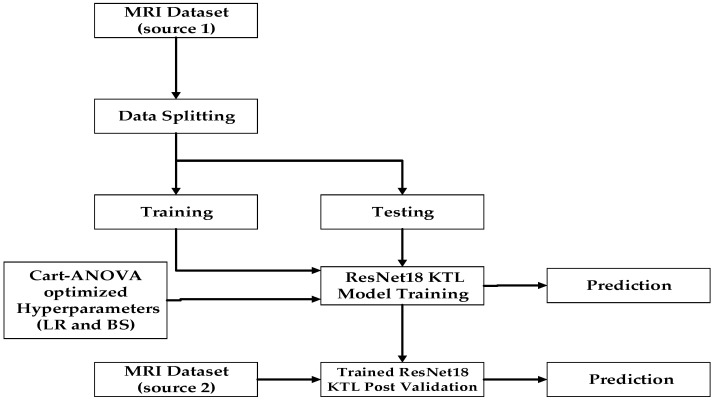
Optimal ResNet18 workflow featuring hyperparameter selection and a comprehensive testing framework for validation.

In this study, we have chosen the ResNet18 KTL architecture due to its performance and notable advantages over more recent deep learning architectures for transfer learning, particularly in the context of medical imaging. Its relatively simple architecture ensures computational efficiency and faster convergence, making it ideal for datasets with limited size and environments with constrained resources. While newer models like DenseNet, EfficientNet, and Vision Transformers offer advanced feature extraction capabilities, they often come with increased computational demands and require larger datasets for optimal fine-tuning. In contrast, ResNet18 achieves high accuracy and sensitivity in brain tumor classification tasks with reduced training complexity, highlighting its effectiveness and practicality for transfer learning applications. Additionally, we introduce a new approach called the CART-ANOVA model. It is an integrated novel approach combining the Cartesian product with ANOVA analysis. CART is the Cartesian product that indicates possible combinations of a learning rate (*LR*) and batch size (*BS*). Furthermore, ANOVA is used to analyze the combinations of hyperparameters (*LR* and *BS*) defined by the Cartesian product to select the hyperparameters that are statistically signified. While compared with the previous studies [15,19,20], ANOVA has been treated as a separate process for the validation of the chosen hyperparameters at the final stage, our method inculcates the ANOVA test uniquely in the framework at the stage of training and testing. This will now allow the model to acquire higher accuracy by choosing the statistically relevant combinations of hyperparameters. The main research contributions of this study are as follows:

- This results in a hyperparameter Cartesian product matrix of the size (7 × 8), which will then be used in training and testing the effect of the hyperparameters *LR* and *BS*, together forming 56 effective two-tuple hyperparameters. Tuples are then used as parameters for 168 runs over the best state-of-the-art pre-trained deep learning model: the ResNet18 model. The effect is further tested through three optimizers: SGDM, Adam, and RMSProp.
- A dataset is produced of 336 model accuracies corresponding to each pair of hyperparameters, (*LR*, *BS*), representing the model performance in brain tumor multiclassification. All hyperparameters could be validated for large impacts individually and interactively by performing statistical ANOVA analysis under simulated results.
- Two separate MRI image datasets are taken, named source 1 and source 2. The source 1 dataset is used for training and testing with an 80:20 ratio, while the source 2 dataset is used for post validation method, as illustrated in Figure 2.

Moreover, many of the transfer learning approaches applied in the previous studies [14,21,22,23] simply trained and tested their models using the same single-source dataset, typically employing an 80:20 split. While this method is convenient, it significantly limits the model’s generalization capability, as it does not adequately test performance on unseen datasets or different modalities. Additionally, the ResNet18 architecture, while effective, can be sensitive to suboptimal hyperparameter settings such as learning rate and batch size, potentially leading to overfitting or underperformance. To address these challenges, the proposed methodology integrates hyperparameter optimization using the CART-ANOVA technique, alongside cross-dataset validation, to enhance both accuracy and robustness. Initially, the source 1 dataset was divided into training and test sets, achieving 99.05% training accuracy for four-class classification and 95% for seven-class classification. On the test dataset (20% split), the model attained 99.65% accuracy for four classes and 98.05% for seven classes. To further demonstrate the model’s robustness, the trained ResNet18 knowledge transfer learning (KTL) model was validated on an entirely different MRI dataset (source 2), yielding 98.78% accuracy for seven classes and 96.78% for four classes. These results confirm the model’s adaptability and effectiveness in handling diverse datasets. The CART-ANOVA-based optimized hyperparameters mitigated the limitations of ResNet18 by reducing the risk of overfitting and ensuring consistent accuracy across different datasets. By combining hyperparameter optimization with cross-dataset validation, the proposed methodology not only addresses the generalization issues prevalent in existing studies but also establishes a more robust framework for brain tumor classification, as illustrated in Figure 2.

This research article is organized as follows: Section 2 provides a comprehensive literature review of brain tumors, focusing on DL methods. Section 3 illustrates the transfer learning technique with preprocessing and augmentation techniques, datasets, pre-trained models, and parameter tuning with the performance assessment. Section 4 explains the analysis and results of the suggested technique, while Section 5 provides the conclusion and future work.

## 2. Literature Review

Over the years, research into improving and developing new optimization techniques has been critical in effectively leveraging the knowledge from pre-trained deep learning models to implement KTL for specific classification tasks. Several studies have addressed the impact of hyperparameters, particularly the learning rate and batch size, on model performance in terms of accuracy and convergence time [14,15,24,25,26,27,28]. However, only a limited number of researchers have conducted statistical analyses for the validation of input hyperparameters to assess the individual and interactive effects of these hyperparameters on network performance [15,29]. Similarly, only a limited number of studies provide tested accuracy on a separate source dataset, as most studies rely on the split mechanism of a single source dataset for both training and testing [30]. Kavin Kumar [31] presents studies to find the best-tested accuracy for brain tumor classification obtaining 96.2% accuracy. However, the study has the limitation that only the impact of the epoch has been discussed without any other hyperparameter discussion, nor is the statistical significance of the parameter included. It also contains the data splitting mechanism using four folds for training and one fold of testing the same dataset that challenges the robustness and generalizability of the model. Kandel and Castelli [25] used the Patch Camelyon histopathologic dataset to identify metastatic tissues in lymph nodes. This dataset falls between CIFAR-10 and ImageNet in size. They evaluated the VGG16 deep learning model using five different batch sizes (16, 32, 64, 128, 256) and two learning rates (0.001, 0.0001). Their findings indicated that both learning rate and batch size significantly impacted network performance, with a strong correlation between the two: higher learning rates paired well with larger batch sizes, while smaller batch sizes worked best with lower learning rates. The authors recommended starting with smaller batch sizes (32 or 64) and lower learning rates. However, their conclusions were based on experiments with limited hyperparameter values, no statistical tests were performed to examine the significance of the interaction between these variables on model performance, and no different source data have been utilized for testing the model. Radiuk et al. [24] investigated the influence of batch size on a pre-trained deep learning model’s performance for image classification tasks using the CIFAR-10 and MNIST datasets. The study tested batch sizes ranging from 16 to 1024 (in powers of two) as well as batch sizes of 50, 100, 150, 200, and 250. For the MNIST dataset, the LeNet architecture was used, while a custom model with five convolutional layers was employed for CIFAR-10. Using the SGD optimizer, they found that a batch size of 1024 yielded the highest accuracy for both datasets, while a batch size of 16 performed the worst. This study highlighted the significant effect of batch size on model performance but did not explore other hyperparameters, such as learning rate, which remained fixed. Another study [26] found that a batch size of 32 is often a suitable default, noting that larger batch sizes can accelerate network processing but may require fewer updates to achieve convergence. Although an appropriate batch size can reduce convergence time, it does not necessarily improve model performance. Meanwhile, the authors of [27] analyzed the effect of batch size on two state-of-the-art models, AlexNet [32] and ResNet [33], using batch sizes ranging from 2 to 32 across three datasets: ImageNet [12], CIFAR-10 [34], and CIFAR-100 [34]. They concluded that batch sizes between 2 and 32 produced favorable results, and smaller batch sizes were generally more robust than larger ones. Usmani et al. [14] introduced a framework for implementing transfer learning for brain tumor classification. They evaluated hyperparameter tuning using a Cartesian product matrix with pairwise combinations of two hyperparameters: learning rate and batch size. Their analysis of 11 pre-trained deep learning models found that ResNet18 architectures performed the best for brain tumor classification. However, the study used only 56 pairs of hyperparameters based on selective values from the literature, making the process less computationally intensive with a further gap of statistical analysis to explore the authentic effects of the learning rate and batch size on performance. Such an analysis could have provided further insights into optimizing these hyperparameters for brain tumor classification. All the above-mentioned studies are limited in terms of dataset generalizability, as they rely on a single-source dataset. Our work systematically addresses the key research gaps identified in existing literature, as illustrated in Figure 2. This framework expands the scope of testing to ensure comprehensive evaluation of the performance, making the detection and classification of the seven classes of brain tumor more authentic and reliable.

## 3. Materials and Methods

### 3.1. Proposed Framework

This section gives a comprehensive description of the suggested framework for using the KTL technique. The steps involve in the classification framework are: (1) Data Collection, which aims to gather and group the required dataset of real MR images; (2) Data Processing, involving preprocessing for image enhancement and resizing; (3) KTL model adaptation; and (4) Post ResNet18 KTL post analysis and validation, which aims to use the pre-trained CNN classification model with layer modification.

The accuracy of the customized KTL model heavily depends on the fine-tuning of selected hyperparameters, specifically batch size and learning rate. For this purpose, the Cartesian-ANOVA technique is employed. The process begins with dataset preparation, comprising MRI images used for training and validation across four and seven distinct brain tumor classifications.

In the next step, the ResNet18-based transfer learning (KTL) model is executed to perform initial experiments, referred to as the TL-run phase. This is followed by the hyperparameter tuning (HPT-run) phase, where statistical evaluations are conducted to determine the most significant combinations of hyperparameters. These combinations are then ranked based on their impact on the model’s accuracy. Using this ranking, the optimal hyperparameters are selected to guide further training and validation phases, seamlessly integrating hyperparameter optimization into the training process.

**Figure 3:**
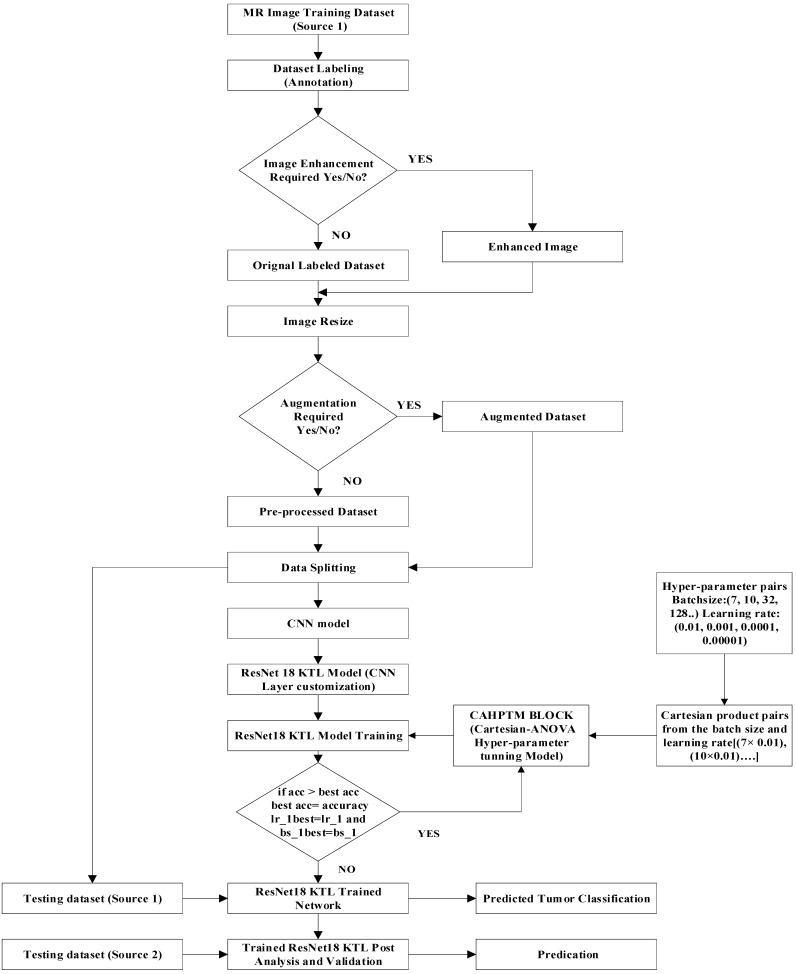
Proposed framework for the best ResNet18 knowledge transfer learning model used for training and validation.

For this study, the source 1 dataset was split, with 80% used for training and 20% reserved for testing. The proposed framework demonstrates high effectiveness in fine-tuning and selecting hyperparameters, producing robust results. The process is summarized in Figure 3 for better understanding.

### 3.2. Datasets

**Figure 4:**
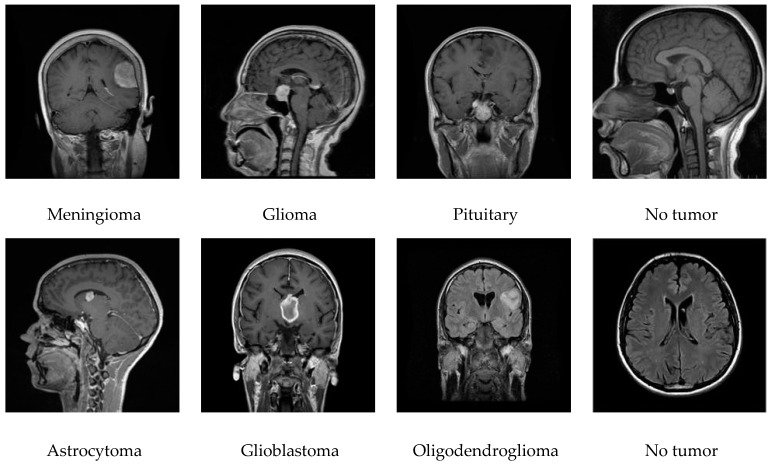
Sample MRI images in the dataset.

In this work, two datasets were used: the source 1 dataset with 3137 images for training and testing that were collected from publicly available datasets Kaggle [35,36] and Radiopaedia [37], and the second dataset of source 2 with 1365 images for validation collected from Radiopaedia [37] and Kaggle [36]. Images were resized to match model requirements, with dimensions of 224 × 224 × 3 for ResNet models. A detailed dataset breakdown is presented in Table 1, while Figure 4 shows some of the sample images stored in the dataset.

### 3.3. Dataset Preprocessing

The presence of noise in brain MRI images results in low classification accuracy [14,38]. Noise reduction was performed using median filtering and sharpening techniques [39], followed by resizing to standard input dimensions of pre-trained models. The typical input size of the pre-trained deep learning model is shown in Table 2 below.

### 3.4. Pre-Trained Deep Knowledge Transfer Learning (KTL) Models

**Figure 5:**
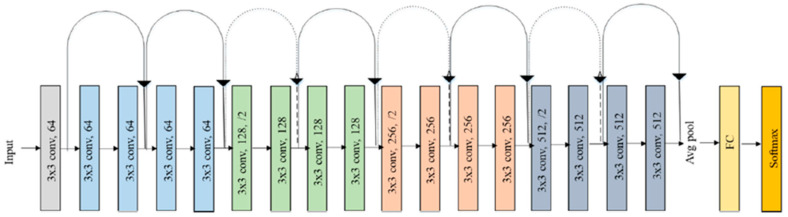
ResNet18 architecture [41].

Recently, several state-of-the-art pre-trained deep transfer learning models for classification have been developed [40]. This became challenging in finding the best possible model for the classification purposes of brain tumors. It was important to select a model that could efficiently transfer knowledge from a natural image dataset to medical imaging. For this research, we have chosen the ResNet18 network because this model proved to be the best architecture for our specific task. ResNet18 maintained its high accuracy with low computationally demanding resources, and thus, it could be an ideal architecture to be fine-tuned and optimized for the classification of brain tumors. This method allowed us to succeed in significant enhancements in performance while systematically analyzing the effects of different hyperparameters. The final fully connected layer was modified to accommodate four and seven classification tasks. Hyperparameter optimization was performed to fine-tune the model for maximal accuracy. The layered architecture of the ResNet18 model is presented in Figure 5.

### 3.5. Transfer Learning Model Optimization and Training

**Figure 6:**
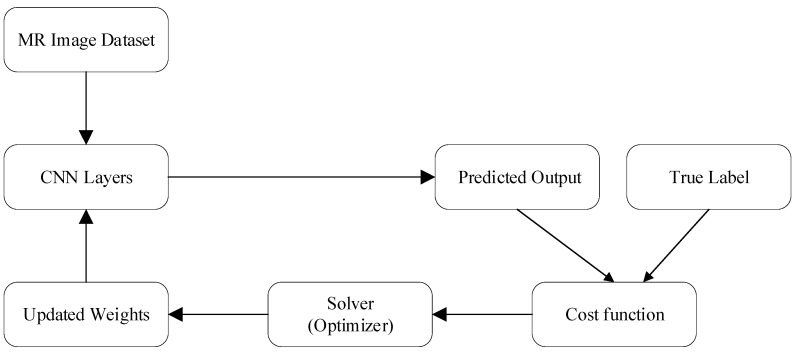
ResNet18 learning process steps.

Learning model optimization reduces cost function value as illustrated in Equation (1) to improve model precision. Convolutional layer filters in CNNs use learnable parameters to extract image features. During training, the solver minimizes loss by iteratively updating parameters based on target and predicted labels, as shown in Figure 6.

(1) $$C=-\frac{1}{n}\sum_{i}^{n}\ln\left(P\left(a^{i}|b^{i}\right)\right)$$

where *n* denotes the number of training images, *b^i^* represents an *i*th image having an *a^i^* label, and $P\left(a^{i}|b^{i}\right)$ is the actual categorization probability. Optimization algorithms like stochastic gradient descent update weights iteratively:

(2) $$W_{l}^{t+1}=W_{l}^{t}+V_{l}^{t+1}$$

where

(3) $$V_{l}^{t+1}=\mu V_{l}^{t}-\gamma^{t}b_{l}\frac{\delta C^{\prime }}{\delta W_{l}}$$

(4) $$\gamma^{t}=\gamma^{\frac{tm}{n}}$$

where $C^{\prime }$ is the mini-batch cost, $W_{l}^{t}$ is the weight at iteration *t*, l is the learning rate, $b_{l}$ is the layer, μ is the momentum in the current iteration that affects previously modified weights, and γ is the scheduling rate that influences the learning rate.

Finally, the ResNet18 model architecture was modified by replacing the fully connected (FC) and classification layers with new layers for four- and seven-class tumor categorization; the classification problem was mathematically defined as

(5) $$f\left(c\right):R^{n}\to R,\;\mathrm{find}\;\left[h1,h2\right]=argmin\;f\left(h1,h2\right),h1,h2ER$$

where $f\left(c\right)$ is the cost function, h1 and h2 are the two hyperparameter (*LR*, *BS*) values required to optimize the cost function with minimum value, while $f\left(h1,h2\right)$ is defined by the following mathematical equation.

(6) $$f\left(h1,h2\right)=\frac{1}{x}\sum_{j=1}^{x}f_{j}\left(h1,h2\right)$$

This integrated approach ensures improved model accuracy and performance through effective knowledge transfer and hyperparameter optimization.

### 3.6. Hyperparameter Tuning with CART-ANOVA Technique

A novel hybrid block termed CART-ANOVA has been introduced that is responsible for optimal hyperparameter selection. The Cartesian product defines different pair combinations of *LR* and *BS*, which are responsible for generating all possible pairs of batch size and learning rate. This work has chosen the Cartesian product matrix of 7 × 8 representing 8 batch sizes and 7 learning rates. One of the possible combinations that we have selected for analysis is *LR* = [0.01, 0.005, 0.001, 0.0005, 0.0001, 0.00005, 0.00001] and *BS* = [2, 4, 7, 8, 10, 16, 32, 64]. These testing combinations are not limited to the selected learning rates and batch sizes used in this study. However, any desired combination of hyperparameters can be tested using this method. The Cartesian product of the sets X and Y can be written as

X × Y = {(x, y)|x ∈ X and y ∈ Y}(7)

For a better understanding of the Cartesian product, it is represented in the following Cartesian matrix:

$$A\times B=\left[\begin{array}{ccccccc} \left(2,\;0.01\right) & \left(2,\;0.005\right) & \left(2,\;0.001\right) & \left(2,\;0.0005\right) & \left(2,\;0.0001\right) & \left(2,\;0.00005\right) & \left(2,\;0.00001\right)\\ \left(4,\;0.01\right) & \left(4,\;0.005\right) & \left(4,\;0.001\right) & \left(4,\;0.0005\right) & \left(4,\;0.0001\right) & \left(4,\;0.00005\right) & \left(4,\;0.00001\right)\\ \left(7,\;0.01\right) & \left(7,\;0.005\right) & \left(7,\;0.001\right) & \left(7,\;0.0005\right) & \left(7,\;0.0001\right) & \left(7,\;0.00005\right) & \left(7,\;0.00001\right)\\ \left(8,\;0.01\right) & \left(8,\;0.005\right) & \left(8,\;0.001\right) & \left(8,\;0.0005\right) & \left(8,\;0.0001\right) & \left(8,\;0.00005\right) & \left(8,\;0.00001\right)\\ \left(10,\;0.01\right) & \left(10,\;0.005\right) & \left(10,\;0.001\right) & \left(10,\;0.0005\right) & \left(10,\;0.0001\right) & \left(10,\;0.00005\right) & \left(10,\;0.00001\right)\\ \left(16,\;0.01\right) & \left(16,\;0.005\right) & \left(16,\;0.001\right) & \left(16,\;0.0005\right) & \left(16,\;0.0001\right) & \left(16,\;0.00005\right) & \left(16,\;0.00001\right)\\ \left(32,\;0.01\right) & \left(32,\;0.005\right) & \left(32,\;0.001\right) & \left(32,\;0.0005\right) & \left(32,\;0.0001\right) & \left(32,\;0.00005\right) & \left(32,\;0.00001\right)\\ \left(64,\;0.01\right) & \left(64,\;0.005\right) & \left(64,\;0.001\right) & \left(64,\;0.0005\right) & \left(64,\;0.0001\right) & \left(64,\;0.00005\right) & \left(64,\;0.00001\right) \end{array}\right]$$

The valid combinations are then checked statistically with the help of the ANOVA block and the Cartesian product combinations. This integrated framework ensured the selection of optimal parameters for training and validation.

**Figure 7:**
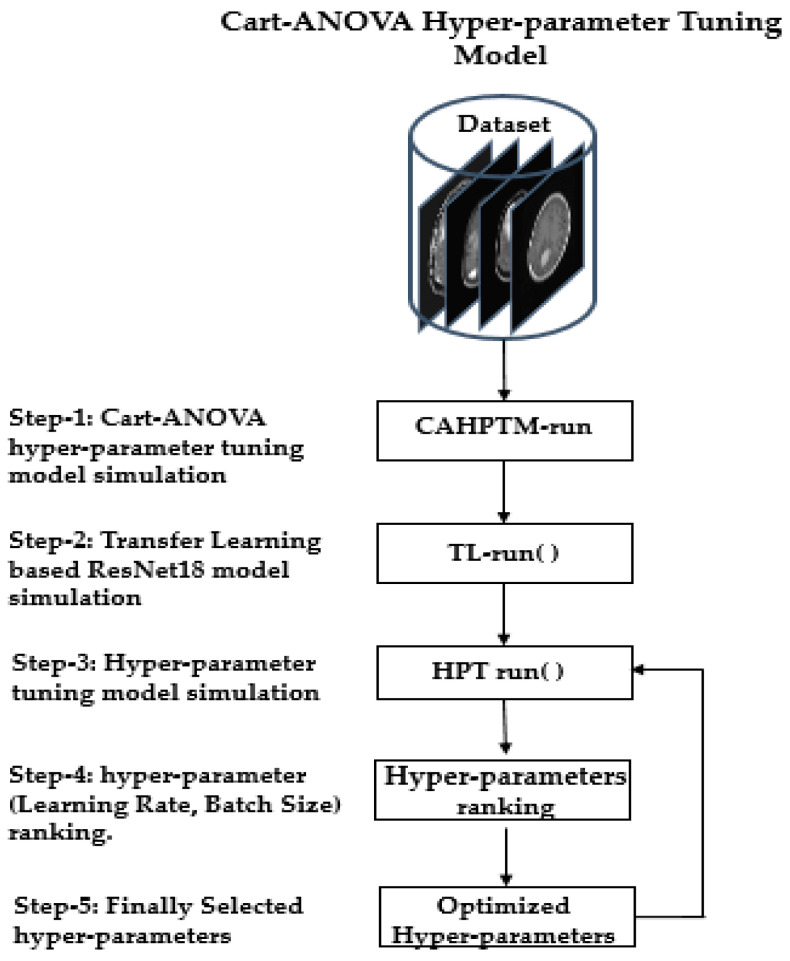
Step of CART-ANOVA-based hyperparameter tuning methodology for the classification of brain tumors.

Figure 7 illustrates the steps involved in the presented method. There are five steps in the process of hyperparameter tuning, as follows:

- Dataset: Images for training and validation, acquired for four and seven distinct classifications of brain tumors.
- TL-run: ResNet18 transfer learning (KTL model) execution of experiments.
- HPT-run: The core block for the statistical evaluation of hyperparameter selection required for the tuning.
- Hyperparameter Ranking: Ranking of the hyperparameters in terms of accuracy evaluation for the running KTL network.
- Hyperparameter: Based on the ranking, hyperparameters are recommended for the new phase of training and validation.

The statistical approach for hyperparameter selection involves two stages: Stage 1 uses ANOVA to assess significant accuracy differences across treatments using the linear model. The ANOVA test linear statistical model is presented in Equation (8):

(8) $$y_{ij}=\mu_{ij}+\in_{ij}$$

where *i* is the hyperparameter combination required for treatment, *j* represents the repetition required for training of the KTL model, *µ_ij_* is the mean accuracy, and ∈*_ij_* is the error term. Stage 2 involves the hyperparameter ranking for training. In the presented model, batch size and learning rate are the recommended hyperparameters. The null hypothesis (H0) and alternative hypothesis (H1) of the experiments are formulated as

Null hypothesis (H0): (*µ*1 = *µ*2 = *µ*3 = *µ*4…*µn*) (9)

Alternative hypothesis (H1): *µi* = *µj* at least for 1 pair of combinations (*i*, *j*) (10)

The ANOVA assessment was made to determine if there is a significant dissimilarity between the means of accuracy, i.e., (*µ*1 *µ*2……*µn*), of the n treatment observed. If the *p*-value > 0.05, H0 is accepted; otherwise, H1 is validated. The selected optimal hyperparameters ensure the highest accuracy for subsequent training and validation phases in this context, the following Algorithm 1 presents the approach for recommending the most suitable hyperparmeters.
**Algorithm 1:** Pseudo code for the proposed CAHPTM modeling***CAHPTM-run ( )***  /* CART-ANOVA-hyper-parameter-tuning-model block*/ *{Hyp learning and tunning ←HPT-run(hyper1, hyper2, results); *   /* hyperparameter learning: Tuning of learning rate and batch size values */ *  hyper 1 ←random learning rate values; hyper 2 ←random batch rate values;* *  run ← number of repetitions;* *  length_lr 1 ←number of LR values; length_bs 1 ←number of BS values;* *  for kl in 1: length_lr 1* *  for kbs in 1: length_bs1* *  for z in 1: run* *  results1[kl, kbs, z] ←TL-run(arguments); }* **TL-run()**   /* Transfer learning simulation block*/ *{    Input: hyperparameters;* *  Set architecture: ResNet18();* *  Set batch size and learning rate: compile();* *  Set data augmentation: image_data_generator() (if required);* *  Set directory: flow_images_from_directory();* *  Return: results of simulation; }* **HPT-run( )**  /* Hyper-parameter tuning block*/ *{    Input: hyper-parameters; results of simulations;*   /*Hyperparameter learning and tunning: Analysis of Variance*/ *  Adjust the ANOVA model: ANOVA();* *  Calculate p-value: p;* *    H1 ← 0;* *  if (p > 0.05) {* *  print(“Not acceptable model”);* *  }else{* *  if (p < 0.05) {* *  H1 ← 1;* *  print(“ANOVA: H1 confirmed”);  }* *else{* *  print(“ANOVA: H0 confirmed”);* *  }  }* **HR-run( )**    /* Hyperparameter Tuning Ranking*/ *{* *  ***if** (*H*_1_ == 1){ *lr_1*, *bs_1   //Select the combination of hyperparameters*
*  model = train model(train data, solver) //Evaluate the model and store the result:* *accuracy = evaluate model(validation data)* *if accuracy > best accuracy then* *{* *best accuracy = accuracy       //mean accuracy * *lr_1best = lr_1 and bs_1best = bs_1 //Selected the best combination of hyperparameters that * *resulted in the best performance* *print (“Recommended hyperparameters:”)* ; *  * } **else**{ *  print(“No difference or not adequate hyperparameter”)* ; *    *} *  ***Return;**

### 3.7. ANOVA (Analysis of Variance)

A two-way ANOVA is used to analyze the effect of *LR* and *BS* on the model performance. Factors that are used in the analysis of the models are represented by the following Equation (11) [42]:

(11) $$Y_{(i,j),k}=\mu +\theta_{i}+\varphi_{j}+\gamma_{i,j}+\epsilon_{(i,j),k}$$

where

*i* represents the *LR* levels (*i* = 1, ……, *a*);
*j* represents the *BS* levels (*j* = 1, ……, *b*);
*k* represents the treatment observations (*k* = 1, ……, *r*; where *r* = (*a* × *b*) at the factor level (*i*, *j*)).

The mean *µ* is represented by Equation (12):

(12) $$\mu =\mu_{..}=\frac{\sum_{i,j}\mu_{i,j}}{ab}$$

where, at the factor level (*i*, *j*), the mean is represented as

(13a) $$\mu_{i}=\frac{\sum_{j}\mu_{i,j}}{b}\;\;(\mathrm{mean}\;\mathrm{at}\;\mathrm{the}\;i\mathrm{th}\;\mathrm{level}\;\mathrm{of}\;\mathrm{LR})\;\mu_{j}=\frac{\sum_{i}\mu_{i,j}}{a}\;\;(\mathrm{mean}\;\mathrm{at}\;\mathrm{the}\;j\mathrm{th}\;\mathrm{level}\;\mathrm{of}\;\mathrm{BS})$$

(13b) $$\theta_{i}=\mu_{i}-\mu \Rightarrow \mu_{i.}=\mu +\theta_{i}\;\;(\mathrm{where}\;\theta_{i}\;\mathrm{is}\;\mathrm{the}\;\mathrm{mean}\;\mathrm{difference}\;\mathrm{at}\;\mathrm{the}\;i\mathrm{th}\;\mathrm{level}\;\mathrm{of}\;\mathrm{LR})\;\varphi_{j}=\mu_{.j}-\mu \Rightarrow \mu_{.j}=\mu +\varphi_{j}\;\;(\mathrm{where}\;\varphi_{j}\;\mathrm{is}\;\mathrm{the}\;\mathrm{mean}\;\mathrm{difference}\;\mathrm{at}\;\mathrm{the}\;j\mathrm{th}\;\mathrm{level}\;\mathrm{of}\;\mathrm{BS})$$

*γ_i,j_* represents the interaction effect between factors *LR* and *BS* and can be defined as

(14) $$\begin{array}{cc} \gamma_{i,j} & =\mu_{i,j}-\left(\mu +\theta_{i}+\varphi_{j}\right)\\ & =\mu_{i,j}-\left(\mu +\left(\mu_{i.}-\mu \right)+\left(\mu_{.j}-\mu \right)\right)\\ & =\mu_{i,j}-\mu_{i.}-\mu_{.j}+\mu \end{array}$$

These equations also explain the connection between the parameters of the factor effects model and the cell means *µ_i_*_,_. Equation (15) represents the overall mean, estimating both the overall mean of all treatments and the mean of the treatments within each group, respectively.

(15) $$\begin{array}{c} \hat{\mu }={\bar{Y}}_{..}=\frac{\sum_{(i,j),r}Y_{(i,j),k}}{abr}\\ {\hat{\mu }}_{i.}={\bar{Y}}_{i..}\;and\;{\hat{\mu }}_{.j}={\bar{Y}}_{.j} \end{array}$$

(16) $${\hat{\theta }}_{i}={\bar{Y}}_{i.}-{\bar{Y}}_{\ldots }\left(\mathrm{where}\;\theta_{i}\;\mathrm{affects}\;\mathrm{LR}\;\mathrm{at}\;\mathrm{the}\;i\mathrm{th}\;\mathrm{level}\right)$$

(17) $${\hat{\varphi }}_{j}={\bar{Y}}_{.j.}-{\bar{Y}}_{\ldots }\left(\mathrm{where}\;{\hat{\varphi }}_{j}\;\mathrm{affects}\;\mathrm{BS}\;\mathrm{at}\;\mathrm{the}\;j\mathrm{th}\;\mathrm{level}\right)$$

${\hat{\gamma }}_{i,j}$ is the interaction effect between factors *LR* and *BS* that can be estimated using Equation (16):

(18) $${\hat{\gamma }}_{i,j}={\bar{Y}}_{i,j.}-{\bar{Y}}_{i..}-{\bar{Y}}_{j.j}+{\bar{Y}}_{\ldots }$$

In the ANOVA table, *SS* presents the sum of the squares, and the total sum of squares for the entire dataset is represented by *SS* (*total*), which is equal to the addition of the sum of squares of *LR*, the sum of squares of *BS*, the sum of squares due to interaction of *LR* and *BS*, and the error sum of squares as presented in Equation (19):

*SS*(*total*) = *SS*(*LR* × *BS*) + *SS*(*LR*) + *SS*(*BS*) + *SS*(*E*) (19)

(20) $$\begin{array}{cc} SS\left(LR\right)=\sum_{(i,j),r}{\hat{\theta }}_{i}^{2} & =\sum_{(i,j),r}{\left({\bar{Y}}_{i..}-{\bar{Y}}_{\ldots .}\right)}^{2}\\ & =rb\sum_{i}{\left({\bar{Y}}_{i..}-\bar{Y}\right)}^{2} \end{array}$$

(21) $$\begin{array}{cc} SS(BS)=\sum_{(i,j),r}{{\hat{\varphi }}_{i}}^{2} & =\sum_{(i,j),r}{\left({\bar{Y}}_{j\cdot }-\bar{Y}..\right)}^{2}\\ & =ra\sum_{j}{\left({\bar{Y}}_{j.}-\bar{Y}\right)}^{2} \end{array}$$

(22) $$\begin{array}{cc} SS(LR\times BS) & =\sum_{(i,j),r}{\hat{\gamma }}_{i,j\;}^{2}\\ & =r\sum_{\left(i,j\right)}{\hat{\gamma }}_{i,j}^{2} \end{array}$$

(23) $$SS\left(E\right)=\sum_{(i,j),r}{\left(Y_{(i,j),r}-{\bar{Y}}_{i,j}\right)}^{2}=\sum_{(i,j),r}e_{(i,j),r}^{2}$$

Another important factor for the ANOVA table is the degree of freedom (*df*). It represents the number of independent values that are free to vary when estimating statistical parameters, like variances. DF plays a crucial role in determining the significance of test statistics.

(24a) $$df_{LR}=\left(a-1\right)$$

(24b) $$df_{BS}=\left(b-1\right)$$

(24c) $$df_{LR\times BS}=\left(a-1\right)\left(b-1\right)$$

(24d) $$df_{E}=ab\left(r-1\right)$$

(24e) $$df_{\mathrm{total}\;}=abr-1$$

The mean square is the sum of the square divided by its corresponding degree of freedom:

(25) $$MS(LR)=SS(LR)/df_{LR}$$

(26) $$MS(BS)=SS(BS)/df_{BS}$$

(27) $$MS(LR\times BS)=\frac{SS(LR\times BS)}{df_{LR\times BS}}$$

(28) $$MS(E)=SS(E)/df_{E}$$

(29) $$MS(\mathrm{total})=SS(\mathrm{total})/df_{\mathrm{total}}$$

(30) $$F_{LR}=\frac{MS(LR)}{MS(E)}\;(\mathrm{hypothesis}\;\mathrm{test}\;\mathrm{for}\;\mathrm{LR}\;\mathrm{effect}\;\mathrm{in}\;\mathrm{two}\text{-}\mathrm{way}\;\mathrm{ANOVA}\;\mathrm{test})$$

(31) $$F_{BS}=\frac{MS(BS)}{MS(E)}\;(\mathrm{hypothesis}\;\mathrm{test}\;\mathrm{for}\;\mathrm{BS}\;\mathrm{effect}\;\mathrm{in}\;\mathrm{two}\text{-}\mathrm{way}\;\mathrm{ANOVA}\;\mathrm{test})$$

(32) $$F_{LR\times BS}=\frac{MS(LR\times BS)}{MS(E)}\;(\mathrm{hypothesis}\;\mathrm{test}\;\mathrm{for}\;\mathrm{LR}\;\mathrm{and}\;\mathrm{BS}\;\mathrm{intersection}\;\mathrm{effect})$$

Significant effects were identified when *p* ≤ 0.05. This analysis confirmed the importance of *LR* and *BS*, as well as their interaction, in optimizing model performance. Table 3 summarizes the key parameters from the two-way ANOVA.

### 3.8. Performance Evaluation Parameters

To verify and evaluate the brain tumor using various techniques. The necessary evaluation criteria in terms of required parameters are explained below.

#### 3.8.1. True and False Values

True values and false (error) values are the fundamental parameters used to evaluate brain tumors. False positive (*FP*), false negative (*FN*), true positive (*TP*), and true negative (*TN*) values are examples of these values. True positive is the term used when the test results are positive and the tumor is also present. A false positive is when a test results in a positive result even if there is not a tumor present. A true negative is when a test results in a negative result and there is not a tumor present, False negatives occur when a test produces a negative result despite the presence of a tumor as shown in Table 4 below.

#### 3.8.2. Other Parameters

*Recall*, *Precision*, *F*1-*Score*, and *Accuracy* were some of the other metrics used to evaluate how well the models performed at classifying brain neoplasms. The following equations used test data to determine the efficiency of the trained model performed overall using the implemented methodology.

(33) $$\mathrm{Recall}=\frac{\mathrm{TP}}{\mathrm{TP}+\mathrm{FN}}$$

(34) $$Precision=\frac{TP}{TP+FP}$$

(35) $$Accuracy=\frac{TP+TN}{TP+FN+TN+FP}$$

(36) $$F1\;Score=2\times \left(\frac{Precision\times Recall}{Precision+Recall}\right)\;$$

## 4. Result Analysis

**Figure 8:**
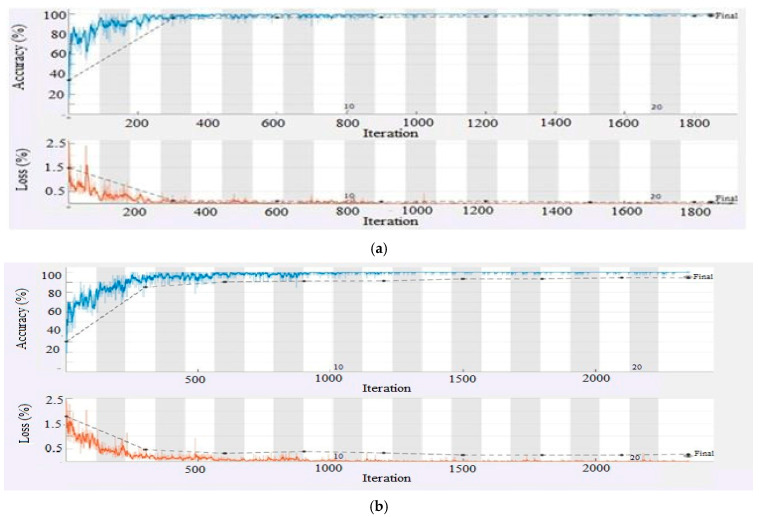
(**a**) Training and validation results using the ResNet18 model optimized with (KTL) with 99.05% validation accuracy for four classification schemes. (**b**) Training and validation results using the ResNet18 model optimized with (KTL) with 95% validation accuracy for seven classification schemes.

**Figure 9:**
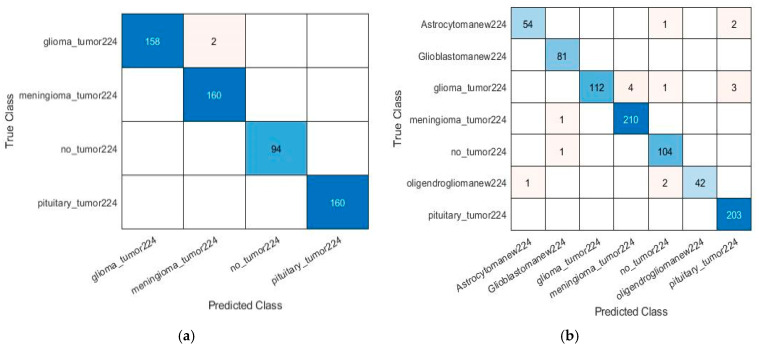
Confusion matrix results using source 1 dataset based on (**a**) classification of four different tumor types by ResNet18 KTL network and (**b**) classification of seven different tumor types by ResNet18 KTL network.

**Figure 10:**
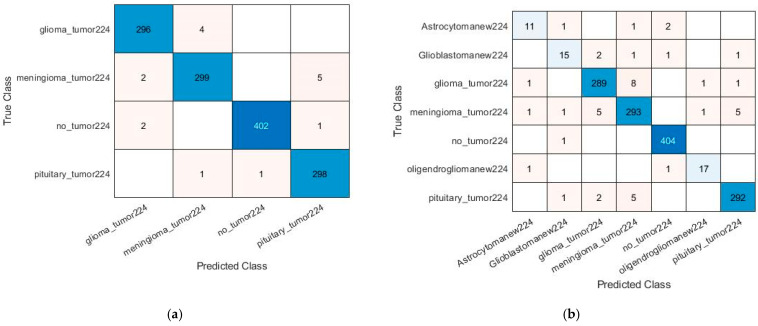
Confusion matrix results using source 2 datasets based on (**a**) classification of four different tumor types by ResNet18 KTL network and (**b**) classification of seven different tumor types by ResNet18 KTL network.

In the present work, simulations along with detailed analysis have been performed using a Hewlett-Packard (HP) high-performance system provided with a core i7 seventh-generation processor, 16 GB RAM, 1TB hard disk, and an NVIDIA GEFORCE GTX 1080 8 GB graphics card, running MATLAB 2020a version 9.8.0.1323502. The equipment was sourced from Karachi, Pakistan. Leveraging the computational capabilities of this setup to efficiently process the brain tumor datasets and perform hyperparameter optimization, this configuration ensured the smooth execution of transfer learning experiments and minimized computational delays, supporting th model’s training and validation processes effectively. The experimental setup achieved a maximum training time of 76 min and a minimum of 17 min, as illustrated in Table 5 and Table 6. Some of the pre-trained models fine-tuned with different hyperparameters were performed and revealed their substantial impact on performance. After extensive simulations, ResNet18 produced exceptional performance, as shown in the training plot in Figure 8, achieving training accuracy of 99.05% (four classes) and 95% (seven classes). The confusion matrices in Figure 9 and Figure 10 highlight the classification performance of the model under different conditions for both four and seven classification schemes. Figure 9 demonstrates near-perfect accuracy, thus achieving the testing accuracy of 99.65% in identifying four classes and 98.05% for seven classes by using the source 1 dataset. In contrast, Figure 10 depicts the model testing with a different dataset (source 2) to evaluate its generalizability. The post-testing results on the unseen dataset also had strong performance confirmation. Specifically, on four-class classification, ResNet18 achieved 98.77% accuracy, while for seven-class classification, it attained 96.77%, which confirms its commendable performance also on the unseen dataset. These results suggest that the model in Figure 9 is optimized for controlled datasets, whereas the model in Figure 10 is more generalizable, effectively handling diverse data with minimal trade-offs in accuracy. From that, there is a clear manifestation of how this model has efficiency in multi-class classification and surpasses other studies. Other metrics, such as precision, specificity, and sensitivity, were tested as shown in Table 5 and Table 6 to reveal that the best model was ResNet18 due to its residual connections, which have been proven to enhance backpropagation and learning of complex hierarchical features. Its lightweight structure and optimal number of layers made it very suitable for smaller datasets.

**Figure 11:**
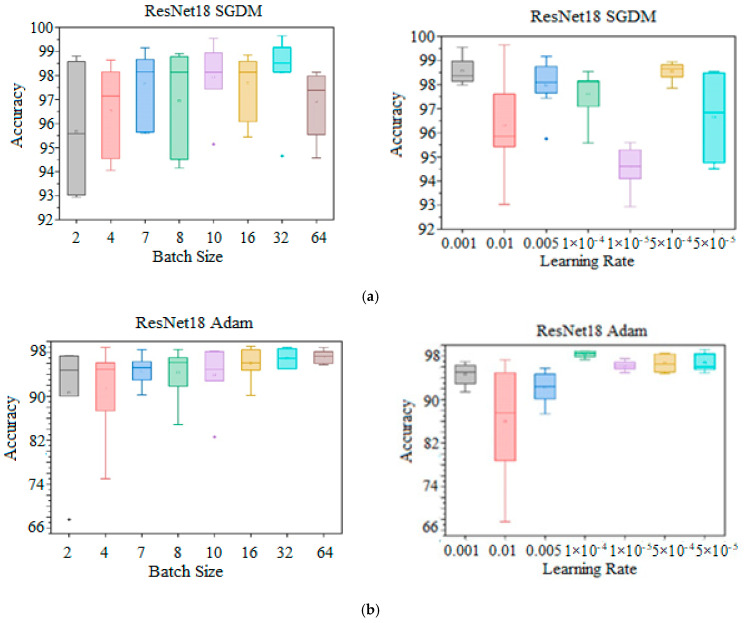

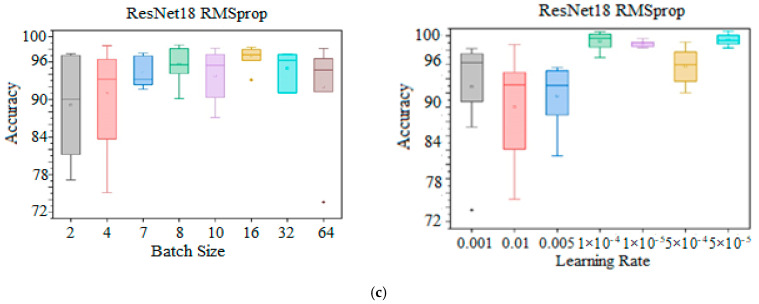
Four-tumor classification statistical performance boxplot. (**a**) KTL ResNet18 with SGDM optimizer: specific *LR*s over wide BR spectrum (right side), and wide *LR* spectrum at specific *BS*s (left side). (**b**) KTL ResNet18 with Adam optimizer: specific *LR*s over wide BR spectrum (right side), and wide *LR* spectrum at specific *BS*s (left side). (**c**) KTL ResNet18 with RMSprop optimizer: specific *LR*s over wide BR spectrum (right side), and wide *LR* spectrum at specific *BS*s (left side).

**Figure 12:**
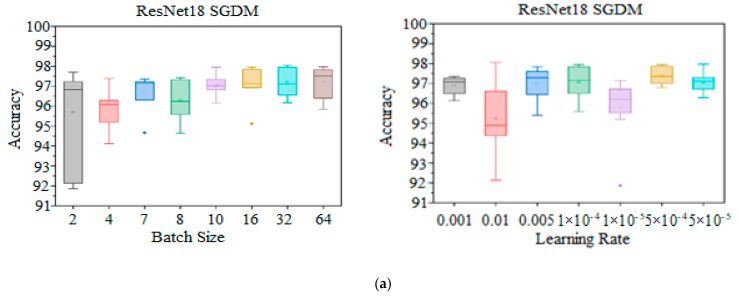

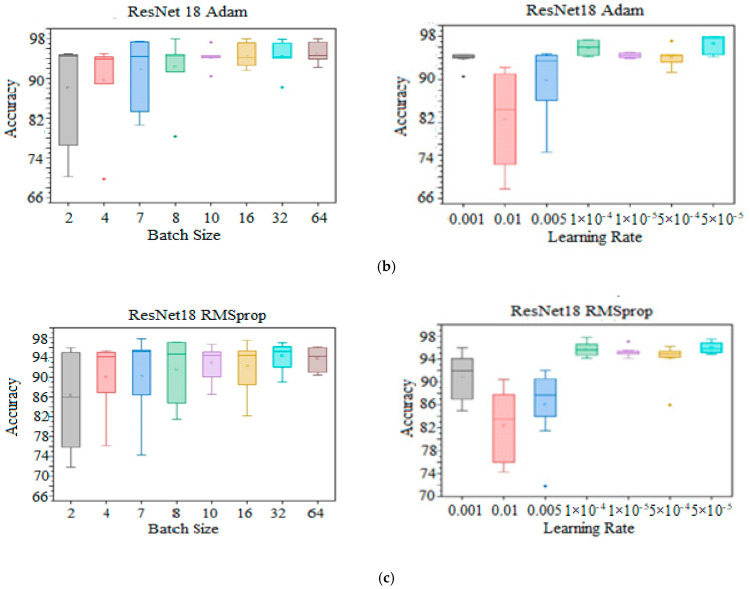
Seven-tumor classification statistical performance boxplot: (**a**) KTL ResNet18 with SGDM optimizer: specific *LR*s over wide BR spectrum (right side), and wide *LR* spectrum at specific *BS*s (left side). (**b**) KTL ResNet18 with Adam optimizer: specific *LR*s over wide BR spectrum (right side), and wide *LR* spectrum at specific *BS*s (left side). (**c**) KTL ResNet18 with RMSprop optimizer: specific *LR*s over wide BR spectrum (right side), and wide *LR* spectrum at specific *BS*s (left side).

Additionally, Figure 11 and Figure 12 display boxplots that highlight the distribution of the obtained findings for the ResNet18 KTL model that was trained by solvers SGDM, Adam, and RMSProp for four and seven brain tumor categorizations, respectively. In both categorizations, each boxplot on the left shows the spectrum of recorded accuracies for the specified range of *LR*s for each particular *BS*, ranging from 2 to 64. Each boxplot on the right side shows the computed accuracies for the specified range of *BS*s at each *LR*, ranging from 0.00001 to 0.01. The five-point plot, including minimum value, lower quartile, median, upper quartile, and maximum points, is represented by each boxplot, and the extreme data points are extended by a whisker to indicate the range of data beyond the IQR. The outlier values are also shown on the plot to indicate the value of data that lie outside the whisker. The boxplot parameters show nonlinear behavior as the *BS*s and *LR*s are increased. Regarding SGDM, a maximum accuracy of 99.65% for four tumor classifications was recorded with *BS* = 32 and *LR* = 0.01, while the highest dispersion spectrum, or maximum value, was observed with *LR* = 0.00005. The natural variability in the accuracy as an outlier occurred at *LR* = 0.005, with an accuracy of 95.75%, whereas the largest dispersion in terms of batch size was seen at *BS* = 02, with an outlier indicating a genuinely rare event occurred at *BS* = 32 and *BS* = 10, achieving accuracies of 94.65% and 95,14%, respectively, as shown in Figure 11a. In contrast, Adam showed the greatest spreading spectrum with *LR* = 0.01 and the maximum point of the whiskers at an accuracy = 99.16% with *LR* = 0.00005 and *BS* = 16, as presented in Figure 11b. On the other hand, RMSProp, the widest spreading spectrum, occurs at *LR* = 0.01 and attains a 96.77% accuracy point as the maximum whisker value. The maximum accuracy of 98.67% was achieved at *LR* = 0.00005 and *BS* = 08, respectively, as depicted in Figure 11c.

Vice versa for the seven-class classification model boxplot, where the highest accuracy achieved was 98.05% at *LR* = 0.01 and *BS* = 32, whereas at *LR* = 0.01 and *BS* = 02, the maximum dispersion spectrum was achieved. Further, the three outlier values as real unique events or the data variability points are also attained at *BS* = 07, *BS* = 16, and *LR* = 0.00001, as depicted in Figure 12a. Comparatively, Adam displayed the highest spreading spectrum with *LR* = 0.01 and the highest whisker value achieved at accuracy = 97.97% with *LR* = 0.00005 and *BS* = 64, as evidenced in Figure 12b. On the other hand, as illustrated in Figure 12c, RMSProp has the widest spreading spectrum at *LR* = 0.01 and achieves a maximum whisker value at 96.77% accuracy. Moreover, at *LR* = 0.00005 with *BS* = 16, the highest accuracy of 97.47% was attained. Decisively, it is clear from the boxplots that changing the *BS*s and *LR*s did not improve or worsen the performance of the model for both four and seven classification schemes in a hierarchical way; instead, there appeared to be a trade-off between the two.

To validate the efficacy of the model, the above statistical analyses are used to validate the results on the dataset using SGDM, Adam, and RMSProp. The boxplot is plotted to illustrate how accuracy varies across different combinations of batch size (*BS*) and learning rate (*LR*). Each boxplot represents a five-point summary: minimum, lower quartile, median (Q2), upper quartile, and maximum with whiskers that extend to show you how the range of data is beyond IQR. Outliers are detected as per the IQR method to take into account rare or extreme events that might affect model performance. This method allowed us to see the variation in data, and how well the patterns or anomalies were established. However, the work has some limitations. Although the observed nonlinear effects for *BS* and *LR* are worthy for predicting the behavior of the model, this still raises concerns about the generalizability of the results to other datasets or models. This shows the importance of additional statistical testing and validation in investigations like ANOVA analysis in future studies to increase the effectiveness of hyperparameter validation that may assist the potential classification.

### 4.1. CART-ANOVA Analysis

In this section, we validate our experimental results through a two-way ANOVA analysis based on the parameter combinations input from Cartesian products. The analysis utilized test accuracy data obtained from Table 7 and the boxplots illustrated in Figure 12. Each accuracy value is maintained for a particular combination of learning rate (*LR*) and batch size (*BS*) for ResNet18 using SGDM, Adam, and RMSProp as solvers. The statistical analysis led to the following conclusions: (1) the application of *LR* played a crucial role in the performance of the model; (2) the application of *BS* also affected the model performance significantly; and (3) an interactive situation between the two *LR*s and *BS*s affected the performance. Furthermore, a data analysis sample from Table 8 is included in the analysis along with a boxplot.

**Figure 13:**
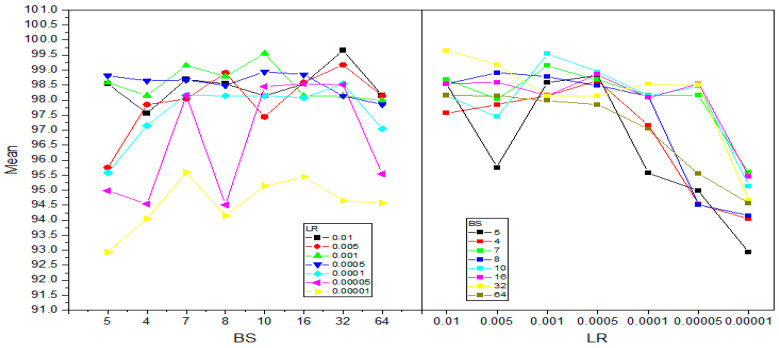
Mean accuracy interaction between learning rate and batch size for four classes.

**Figure 14:**
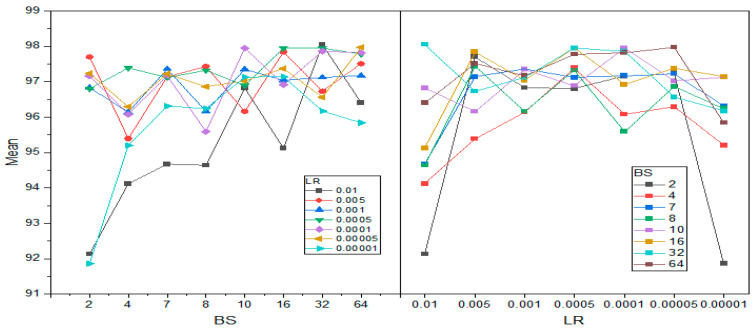
Mean accuracy interaction between learning rate and batch size for seven classes.

In Table 8, columns represent *LR*, and rows represent *BS*. The first three rows are ResNet18 (SGDM), ResNet18 (Adam), and ResNet18 (RMSprop) for *BS* = 2, and the last three rows indicate their performance with *BS* = 4. The response values reflect the accuracy of each model at a certain combination of *LR* and *BS*. Table 9 and Table 10 summarize the outcome of the analysis using ANOVA. The corresponding *p*-values for the parameter “Prob > F” in *LR*, *BS*. Hence, these *p*-values indicate that both *LR*s and *BS*s independently impacted model performance. A series of multiple comparison tests were also run to decide whether there is any significant performance difference between all pairs of *LR*s. This analysis helped identify the notable effect of increasing or decreasing *LR*s on model accuracy. Figure 13 and Figure 14 show the difference in mean values for both four and seven classes concerning learning rate and batch size variations.

The plot in Figure 13 and Figure 14 shows the variation in accuracy and presents the performance of the batch size variation concerning different learning rates for four and seven classes, respectively. For example, most of the learning rates seem to have higher mean accuracies at both batch sizes of 16 and 32. This variability supports the ANOVA analysis, which aims to identify significant differences between groups. In the same manner as on the left-hand-side plot, there is also variability showing at different learning rates. This aligns with the ANOVA findings, which indicated significant differences across groups.

In Table 11 and Table 12, columns 1 and 2 list the pairs of *LR*s being compared. Column 3 is the calculated difference in group means; columns 5 and 6 are the lower and upper limits of the 95% confidence interval for the true mean difference. Column 4 reports the *p*-value from the hypothesis test. The last column represents significance by using H0 information. Table 8 explicitly shows that differences in the group mean of more than one unit were associated with *p*-values smaller than 0.05. Given the extremely small *p*-values in Table 8, model performance differed significantly when applied to different *LR*s. Hence, the values of *LR*s notably influenced model performance. However, another set of multiple comparisons was conducted by the CART-ANOVA model to determine the effect of *BS* on performance. Table 13 and Table 14 show the results in terms of mean accuracies for eight different *BS*s. In this case, very small *p*-values (<0.05) denote that group means were not equal and, therefore, the batch size significantly impact the model performance.

Table 15 summarizes a ranking of many combinations of hyperparameters obtained with their corresponding mean accuracy. Mean accuracy serves as the main evaluative criterion for each separate combination of learning rates and batch sizes, as is noted under the hyperparameter column. The goal is to obtain the optimal hyperparameters that obtain the highest accuracy on the dataset.

### 4.2. K-Fold Cross-Validation

**Figure 15:**
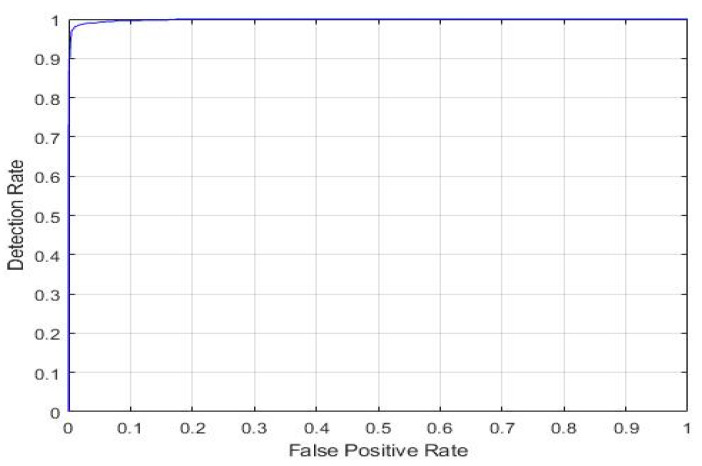
KTL ResNet-18 K-fold cross-validation result for four classes, achieving an AUC of 99.48%.

**Figure 16:**
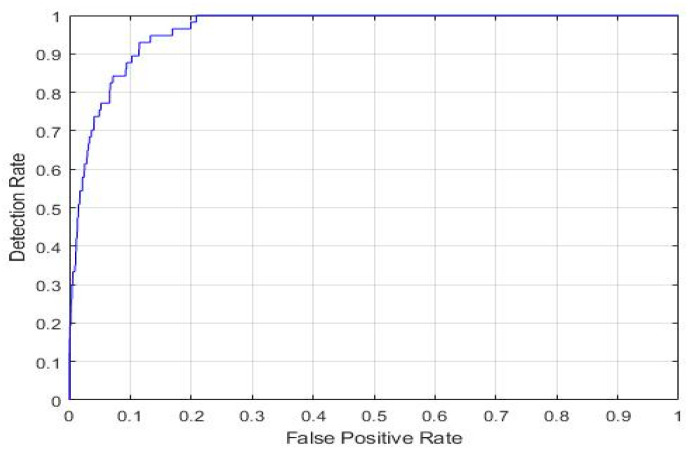
KTL ResNet-18 K-fold cross-validation result for seven classes, achieving the AUC of 96.28%.

This work aimed to validate the performance of our best knowledge-based transfer learning ResNet18 model using hyperparameter tuning. The validation method known as the K-fold cross-validation technique was utilized. It is particularly useful when the dataset is limited and you want to make efficient use of the available data for both training and testing. We partitioned the data into five folds for testing and training. In 5-fold iterations, ResNet18 CNN training and validation were carried out. Four folds are used for training, while one fold is used for testing every iteration. Every iteration’s accuracy was measured, and the model’s accuracy was determined as the average accuracy over all iterations. Data stratification is necessary for accuracy that is widely recognized. The process of stratification involves rearranging the data in each fold so that they are a valid representation of the data overall. The following Figure 15 and Figure 16 show the K-fold graph for both classes. The K-fold cross-validation result proves the efficiency of our KTL model, achieving average AUC values of 99.48% and 96.28% with average accuracies of 97.35% and 91% for the four and seven classifications, respectively.

**Figure 17:**
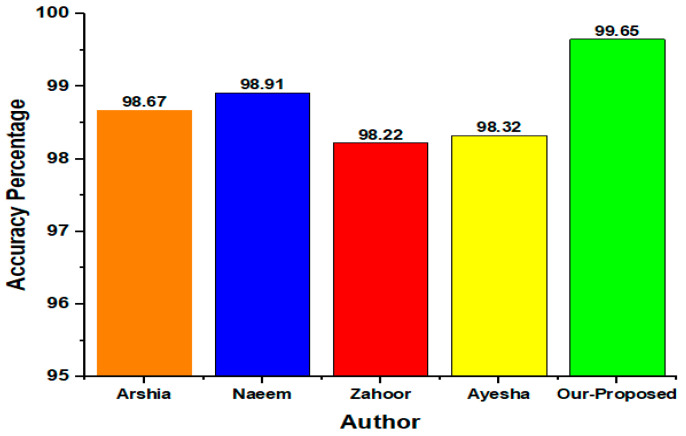
Comparison of accuracy using the proposed and state-of-the-art techniques.

The results of the presented model when compared with the state-of-the-art (SOTA) techniques that have been illustrated in recent studies [6,7,14,15,16,17,44,45]. It was found that the presented model displays remarkable classification results, as shown in Figure 17. This shows that, although the other frameworks are generally effective in their domains, specifically this KTL Resnet18 framework offers a precise distinction between complex tumor categories, further underscoring the potential of our proposed framework in clinical applications. A comparison of some state-of-the-art methods is illustrated in Table 16.

Expanding further what these results mean in a clinical setting, the enhanced KTL classification can offer an accurate way to classify brain tumor types, which could support more precise diagnostic imaging and thus guide treatment for higher patient outcomes. Such an improved distinction of tumor types, as well as the sub-classifications of gliomas such as glioblastoma, astrocytoma, and oligodendroglioma, to name a few, allows clinicians to tailor treatment plans more efficiently with better-targeted therapies translating into superior survival rates. This technique could result in fewer invasive diagnostic procedures being required, providing a non-invasive alternative that uses the high level of information available from MRI images. Nevertheless, in actual clinical practice, this methodology faces serious issues. The invariance of the model maintains its high accuracy across different healthcare facilities with potentially very diverse MRI machines and imaging protocols. Moreover, incorporating this sophisticated AI method in the context of current clinical workflows needs to be carried out prudently by training clinicians and adjusting existing systems to integrate these new tools. Addressing these challenges will likely necessitate partnerships with clinicians to validate and iterate the model, as well as protocols of good practice and intuitive design focused on encouraging adoption in clinical settings.

### 4.3. Enhanced Discussion Section

To improve the discussion section, we have adapted the equator network STARD checklist. The purpose of this is to critically evaluate the results while ensuring alignment with the checklist guidelines. Below Table 17 present some of the most appropriate self-assessments based on the STARD checklist; it compares the checklist requirements against the current study’s results and discussion, which reflects the areas addressed.

## 5. Conclusions and Future Work

This study introduced a novel knowledge transfer learning (KTL) framework that achieved significant advancements in enhanced brain tumor classifications. The framework’s novelty lies in its ability to classify tumors into seven categories, and in the introduction of a new CART-ANOVA block that optimizes hyperparameters. This block selects appropriate learning rate (*LR*) and batch size (*BS*) pairs based on ANOVA tests performed on the Cartesian product of these parameters. Beyond ANOVA, a post-ANOVA analysis, specifically the Honest Significant Difference (Tukey’s HSD) test, was employed to conduct pairwise comparisons among *LR* and *BS* groups. This additional statistical validation enabled the identification of significant differences across hyperparameter groups, supporting the rejection of the null hypothesis and underscoring the critical role of these parameters in optimal model selection. As well as this rigor in hyperparameter tuning and cross-dataset validation, the framework includes a post-test analysis step, where the model is trained and tested on a primary dataset (source 1), followed by validation on a separate dataset (source 2) to ensure generalizability and robustness. ResNet18 outperformed other models with a testing accuracy of 99.65% and 98.05% across four- and seven-class tasks on the primary dataset, and 98.77% and 96.77% on the secondary dataset. With such promising outcomes, our framework has set a new benchmark in precision, accuracy, and sensitivity in CNN-based brain tumor detection. To address broader applicability, the proposed framework has the potential to extend beyond brain tumor classification to other medical imaging tasks. Its adaptability to optimize hyperparameters and validate performance across datasets makes it a valuable tool for applications such as organ segmentation, lesion detection, and other disease classification tasks, setting a benchmark in precision, accuracy, and sensitivity in CNN-based medical imaging analysis.

Future work should focus on incorporating multi-modal imaging, such as CT and PET scans, and combining clinical data like patient history to provide more comprehensive diagnoses. Additionally, developing explainable AI models, exploring hybrid architectures, and utilizing semi-supervised learning and data augmentation can enhance the model’s robustness and generalizability. Collaboration between machine learning experts and clinicians will be essential to align these models with real-world clinical workflows.

## Figures and Tables

**Table 1:** Dataset detail for both seven- and four-class classification datasets.

Tumor Types	Dataset Details/Specs for Seven Classes		Dataset Details/Specs for Four Classes	
	Dataset Source 1	Dataset Source 2	Dataset Source 1	Dataset Source 2
Glioma	826	300	826	300
Meningioma	822	306	822	306
Pituitary	827	300	827	300
No tumor	395	405	395	405
Glioblastoma	165	20	---	---
Astrocytoma	57	15	---	---
Oligodendroglioma	45	19	---	---
Total	3137	1365	2870	1311

**Table 2:** Implemented DL network input image size specifications.

DL Model Name	Input Image Size
GoogleNet, VGG19, VGG16, ResNet101, ResNet50, and ResNet18	224 × 224 × 3
Squeeze-Net and Alex-Net	227 × 227 × 3
Inception V3	229 × 229 × 3

**Table 3:** The two-way ANOVA table [43].

Source	*SS*	*df*	*MS*	*F*
Columns (*LR*)	*SS* (*LR*)	(*a* − 1)	*MS*(*LR*)	$\frac{MS(LR)}{MS(E)}$
Rows (*BS*)	*SS* (*BS*)	(*b* − 1)	*MS*(*BS*)	$\frac{MS(BS)}{MS(E)}$
Interaction (*LR* × *BS*)	*SS* (*LR* × *BS*)	(*a* − 1)(*b*− 1)	*MS*(*LR* × *BS*)	$\frac{MS(LR\times BS)}{MS(E)}$
Error	*SS* (*E*)	(*r* − 1)	(*EE*)	
Total	*SS* (*total*)	*abr* − 1	(*total*)	

**Table 4:** True and false values.

Test	Tumor	No Tumor
“Positive Test”	*TP*	*FP*
“Negative Test”	*FN*	*TN*

**Table 5:** Comparative analysis of pre-trained network for brain tumor four-class classification model.

Model Name	Learning Rate	Batch Size	Epoch	Optimizer	Validation Accuracy	Testing Accuracy	*Precision*	*Recall*	*F*1-*Score*	Training Time
AlexNet	0.001	32	15	SGDM	96.52	96.16	96.56	96.57	96.56	00:17:04
GoogleNet	0.0001	10	16	Adam	96.86	98.25	98.43	98.45	98.44	00:19:04
ResNet-18	0.01	32	21	SGDM	99.05	99.65	99.69	99.69	99.69	00:21:05
ResNet-50	0.01	32	20	SGDM	97.56	98.60	98.75	98.77	98.76	00:28:10
ResNet-101	0.01	32	20	SGDM	98.61	99.47	99.53	99.54	99.53	00:55:02
VGG-19	0.0001	7	08	SGDM	97.91	99.30	99.37	99.38	99.37	00:25:11
VGG-16	0.0001	7	10	SGDM	96.86	98.43	98.59	98.63	98.61	00:18:20
SqueezeNet	0.0001	32	36	SGDM	95.47	97.38	97.65	98.02	97.83	00:15:05
Inception-V3	0.0001	10	11	RMS prop	95.82	97.91	98.01	98.13	98.07	01:04:05

**Table 6:** Comparative analysis of pre-trained network for brain tumor seven-class classification model.

Model Name	Learning Rate	Batch Size	Epoch	Optimizer	Validation Accuracy	Testing Accuracy	*Precision*	*Recall*	*F*1-*Score*	Training Time
AlexNet	0.001	32	35	SGDM	89.14	90.14	88.30	89.22	88.76	00:28:10
GoogleNet	0.0001	10	100	Adam	90.38	90.02	88.44	87.79	88.13	00:29:58
ResNet-18	0.01	32	21	SGDM	95.00	98.05	98.25	97.14	97.69	00:31:55
ResNet-50	0.01	32	21	SGDM	91.05	94.89	96.05	96.22	96.14	00:39:12
ResNet-101	0.01	32	17	SGDM	92.65	93.67	95.86	96.25	96.05	01:07:03
VGG-19	0.0001	7	09	SGDM	90.10	90.87	87.81	90.05	88.91	00:36:55
VGG-16	0.0001	7	10	SGDM	91.37	91.24	90.62	88.35	89.47	00:29:05
SqueezeNet	0.0001	32	31	SGDM	91.37	92.33	91.93	89.13	90.51	00:25:40
Inception-V3	0.0001	10	06	RMS prop	91.05	93.31	92.35	92.13	92.23	01:16:55

**Table 7:** Accuracy chart obtained for four and seven class categories using ResNet18 with three different solvers.

ResNet18 SGDM for four classes								ResNet18 SGDM for seven classes							
*BS*/*LR*	0.01	0.005	0.001	0.0005	0.0001	0.00005	0.00001	*BS*/*LR*	0.01	0.005	0.001	0.0005	0.0001	0.00005	0.00001
2	93.03	95.21	95.64	98.78	97.82	96.08	99.65	2	92.13	97.7	96.83	97.57	97.16	97.23	91.86
4	95.75	97.85	98.04	98.91	97.44	98.59	99.17	4	94.12	95.39	96.14	97.39	96.08	96.29	95.2
7	98.96	98.15	98.95	98.79	99.55	98.14	98.14	7	94.67	97.14	97.69	97.12	97.17	97.22	96.31
8	98.81	98.64	98.67	98.49	98.94	98.85	98.14	8	94.64	97.43	96.16	97.32	95.59	96.86	96.24
10	95.58	97.15	99.13	98.14	98.14	98.08	98.54	10	96.82	96.16	97.81	96.89	97.95	97.02	97.13
16	94.98	94.54	98.15	94.51	98.45	98.54	98.51	16	95.12	97.84	97.04	97.95	96.92	97.95	97.14
32	99.65	94.05	95.59	94.15	95.14	95.44	94.65	32	98.05	96.73	97.12	97.95	97.86	97.95	96.17
64	93.03	95.21	95.64	95.64	97.82	96.08	94.25	64	96.4	97.51	97.17	97.78	97.82	97.97	95.84
ResNet18 ADAM for four classes								ResNet18 ADAM for seven classes							
*BS*/*LR*	0.01	0.005	0.001	0.0005	0.0001	0.00005	0.00001	*BS*/*LR*	0.01	0.005	0.001	0.0005	0.0001	0.00005	0.00001
2	67.56	90.02	91.39	94.75	97.36	95.88	97.41	2	70.25	76.57	90.55	94.52	94.72	94.93	94.79
4	75.01	87.36	93.03	94.91	98.08	96.12	94.96	4	69.75	88.91	93.89	92.26	96.94	94.32	94.97
7	90.23	92.98	94.02	95.21	98.47	95.42	96.32	7	80.63	83.32	94.07	94.40	97.36	97.48	94.98
8	84.85	91.82	96.17	97.05	98.51	95.63	96.18	8	78.33	94.54	94.53	91.30	94.83	97.94	94.63
10	82.58	94.38	92.81	98.26	98.14	94.92	96.34	10	90.42	94.45	94.62	94.32	97.27	94.19	94.12
16	94.78	90.19	96.08	98.47	97.60	98.60	96.08	16	91.62	92.60	94.20	94.18	97.44	97.95	94.31
32	95.02	94.99	96.95	98.47	98.25	98.69	95.21	32	88.18	94.31	94.62	97.13	97.08	97.89	94.03
64	97.31	95.75	96.45	95.98	98.40	98.14	97.50	64	92.23	94.76	94.31	94.59	97.32	97.97	93.89
ResNet18 RMSprop for four classes								ResNet18 RMSprop for seven classes							
*BS*/*LR*	0.01	0.005	0.001	0.0005	0.0001	0.00005	0.00001	*BS*/*LR*	0.01	0.005	0.001	0.0005	0.0001	0.00005	0.00001
2	77.15	81.21	85.22	90.02	95.66	97.29	97.02	2	75.83	71.82	84.97	85.97	95.97	95.02	95.02
4	75.13	83.68	93.22	92.95	97.90	96.31	96.41	4	76.12	86.89	87.56	95.35	96.83	94.82	95.04
7	91.63	93.11	92.35	93.21	94.94	97.40	96.95	7	74.28	86.45	86.42	95.42	97.78	95.23	95.51
8	94.12	90.14	95.53	95.23	98.12	97.73	97.62	8	84.73	81.46	90.85	94.67	97.12	96.71	97.06
10	87.12	91.04	95.44	90.32	97.16	98.16	97.02	10	86.53	90.07	93.04	95.12	94.42	96.68	94.69
16	96.77	93.11	96.21	97.12	98.33	97.98	97.26	16	82.19	88.49	93.13	94.42	95.25	97.47	95.34
32	91.03	91.05	95.25	96.24	97.22	97.18	96.58	32	89.04	92.05	95.02	96.21	96.04	96.98	95.21
64	91.22	93.57	73.61	94.69	98.13	96.52	96.33	64	90.43	91.02	95.98	94.23	94.89	96.16	94.12

**Table 8:** The sample dataset established for ANOVA statistics.

ResNet18 SGDM for Four Classes								ResNet18 SGDM for Seven Classes						
*BS*/*LR*	0.01	0.005	0.001	0.0005	0.0001	0.00005	0.00001	0.01	0.005	0.001	0.0005	0.0001	0.00005	0.00001
2	93.03	95.21	95.64	95.64	97.82	96.08	99.65	92.13	97.7	96.83	96.8	97.16	97.23	91.86
	67.56	90.02	91.39	94.75	97.36	95.88	97.41	70.25	76.57	90.55	94.52	94.72	94.93	94.79
	77.15	81.21	85.22	90.02	95.66	97.29	97.02	75.83	71.82	84.97	85.97	95.97	95.02	95.02
4	95.75	97.85	98.04	98.91	97.44	98.59	99.17	94.12	95.39	96.14	97.39	96.08	96.29	95.2
	75.01	87.36	93.03	94.91	98.91	96.12	94.96	69.75	88.91	93.89	92.26	94.21	94.32	94.97
	75.13	83.68	93.22	92.95	98.58	96.31	96.41	76.12	86.89	87.56	95.35	94.17	94.82	95.04

**Table 9:** The ANOVA statistics outcome for four classes.

Source of Variation	*SS*	*df*	*MS*	*F*	*p*-Value	*F* Crit
*BS*	20.60937	7	2.944196	4.053475	0.00178	2.23707
*LR*	101.9806	6	16.99677	23.4006	6.23 × 10^−12^	2.323994
Error	30.50623	42	0.726339			
Total	153.0962	55				

**Table 10:** The ANOVA statistics outcome for seven classes.

Source of Variation	*SS*	*df*	*MS*	*F*	*p*-Value	*F* Crit
*BS*	18.0441	7	2.577728	2.602535	0.025198	2.23707
*LR*	15.05439	6	2.509065	2.533212	0.03489	2.323994
Error	41.59966	42	0.990468			
Total	74.69816	55				

**Table 11:** The two-way ANOVA with multiple comparisons of *LR* (column-wise) means for four classes.

Group A	Group B	Mean Difference (A)	*p*-Value	Lower Limit	Upper Limit	Reject Null Hypothesis
1	2	3.8418	0.03	−1.6171	4.56625	True
1	3	5.3118	0.03	−0.1471	5.79125	True
1	4	7.15125	0.03	1.69245	7.324075	True
1	5	7.72125	0.03	2.26245	7.799075	True
1	6	6.00555	0.03	0.54665	6.369375	True
1	7	6.00555	0.03	0.54665	6.369375	True
2	3	1.47	0.03	−3.9888	2.5897	True
2	4	3.30945	0.03	−2.14945	4.122575	True
2	5	3.87945	0.03	−1.57945	4.597575	True
2	6	2.16375	0.03	−3.29505	3.167825	True
2	7	2.16375	0.03	−3.29505	3.167825	True
3	4	1.83945	0.03	−3.61945	2.897575	True
3	5	2.40945	0.03	−3.04945	3.372575	True
3	6	0.69375	0.9984	−4.76505	1.942825	False
3	7	0.69375	0.9984	−4.76505	1.942825	False
4	5	0.57	0.9995	−4.8888	1.8397	False
4	6	−1.1457	0.9767	−6.6046	0.41	False
4	7	−1.1457	0.9767	−6.6046	0.41	False
5	6	−1.71555	0.854	−7.17445	−0.06493	False
5	7	−1.71555	0.854	−7.17445	−0.06493	False
6	7	0	1	−5.4588	1.3647	False

**Table 12:** The two-way ANOVA with multiple comparisons of *LR* (column-wise) means for seven classes.

Group A	Group B	Mean Difference (A-B)	*p*-Value	Lower Limit	Upper Limit	Reject Null Hypothesis
1	2	1.99305	0.03	−1.36805	2.501175	True
1	3	1.99875	0.03	−1.36225	2.505875	True
1	4	2.4957	0.03	−0.8654	2.92	True
1	5	1.74	0.03	−1.621	2.29025	True
1	6	1.87695	0.03	−1.48415	2.404375	True
1	7	−0.73875	0.0970	−4.09975	0.224625	True
2	3	0.00555	0.001	−3.35555	0.844925	True
2	4	0.5025	0.0096	−2.8585	1.259	True
2	5	−0.2532	0.0010	−3.6143	0.6293	True
2	6	−0.11625	1	−3.47725	0.743375	False
2	7	−2.73195	0.0257	−6.09305	−1.43632	True
3	4	0.49695	0.9963	−2.86415	1.254375	False
3	5	−0.25875	0.9999	−3.61975	0.624625	False
3	6	−0.1218	1	−3.4829	0.73875	False
3	7	−2.7375	0.0252	−6.0985	−1.441	True
4	5	−0.7557	0.9673	−4.1168	0.21055	False
4	6	−0.61875	0.9881	−3.97975	0.324625	False
4	7	−3.23445	0.0045	−6.59555	−1.85507	True
5	6	0.13695	1	−3.22415	0.954375	False
5	7	−2.47875	0.0567	−5.83975	−1.22538	False
6	7	−2.6157	0.0373	−5.9768	−1.33945	True

**Table 13:** The two-way ANOVA with multiple comparisons of *BS* (row-wise) means for four classes.

Group A	Group B	Mean Difference (A-B)	*p*-Value	Lower Limit	Upper Limit	Reject Null Hypothesis
1	2	2.52	0.01	−7.23225	2.781225	True
1	3	5.2287	0.01	−4.29793	4.699888	True
1	4	3.2658	0.01	−6.4244	3.3095	True
1	5	5.6913	0.01	−3.79683	5.027588	True
1	6	3.2658	0.01	−6.4244	3.3095	True
1	7	5.6958	0.01	−3.7919	5.03075	True
1	8	−0.5613	1	−10.5704	0.598637	False
2	3	2.7087	0.01	−7.02793	2.914888	True
2	4	0.7458	1	−9.1544	1.5245	False
2	5	3.1713	0.01	−6.52683	3.242588	True
2	6	0.7458	1	−9.1544	1.5245	False
2	7	3.1758	0.01	−6.5219	3.24575	True
2	8	−3.0813	0.9913	−13.3004	−1.18636	False
3	4	−1.9629	0.9995	−12.0889	−0.39414	False
3	5	0.4629	1	−9.46087	1.324112	False
3	6	−1.9629	0.9995	−12.0889	−0.39414	False
3	7	0.4671	1	−9.45638	1.327113	False
3	8	−5.79	0.7851	−16.2348	−3.10502	False
4	5	2.4258	0.01	−7.3344	2.7145	True
4	6	0	1	−9.96225	0.996225	False
4	7	2.43	0.01	−7.32975	2.717475	True
4	8	−3.8271	0.97	−14.1084	−1.71464	False
5	6	−2.4258	0.998	−12.5904	−0.72202	False
5	7	0.0042	1	−9.95785	0.999225	False
5	8	−6.2529	0.7137	−16.7364	−3.43289	False
6	7	2.43	0.01	−7.32975	2.717475	True
6	8	−3.8271	0.97	−14.1080	−1.71464	False
7	8	−6.2571	0.713	−16.7409	−3.43589	False

**Table 14:** The two-way ANOVA with multiple comparisons of *BS* (row-wise) means for seven classes.

Group A	Group B	Mean Difference (A-B)	*p*-Value	Lower Limit	Upper Limit	Reject Null Hypothesis
1	2	2.8935	1	−2.52185	1.884575	FALSE
1	3	23.4	0.0001	21.45946	14.33088	TRUE
1	4	14.55975	0.0001	11.12109	8.965347	TRUE
1	5	30.89025	0.0001	30.21879	18.87705	TRUE
1	6	31.0815	0.0001	30.44236	18.99313	TRUE
1	7	34.49025	0.0001	34.42879	21.06205	TRUE
1	8	34.65	0.0001	34.61571	21.15901	TRUE
2	3	20.5065	0.0001	18.07549	12.57469	TRUE
2	4	11.6685	0.0001	7.740025	7.210512	TRUE
2	5	27.99675	0.0001	26.83501	17.12085	TRUE
2	6	28.19025	0.0001	27.06129	17.2383	TRUE
2	7	31.59675	0.0001	31.04501	19.30585	TRUE
2	8	31.7565	0.0001	31.23174	19.40282	TRUE
3	4	−8.84025	0.9986	−16.2439	−5.23715	FALSE
3	5	7.49025	0.0001	2.853794	4.674547	TRUE
3	6	7.6815	0.0001	3.077363	4.790631	TRUE
3	7	11.09025	0.0001	7.063794	6.859547	TRUE
3	8	11.25	0.0001	7.250712	6.956506	TRUE
4	5	16.32825	0.0001	13.18926	10.03873	TRUE
4	6	16.52175	0.0001	13.41563	10.15617	TRUE
4	7	19.92825	0.0001	17.39926	12.22373	TRUE
4	8	20.09025	0.0001	17.58879	12.32205	TRUE
5	6	0.1935	1	−5.67935	0.245825	FALSE
5	7	3.6	1	−1.69554	2.313381	FALSE
5	8	3.75975	1	−1.50891	2.410347	FALSE
6	7	3.4065	1	−1.92201	2.195944	FALSE
6	8	3.5685	1	−1.73247	2.294263	FALSE
7	8	0.15975	1	−5.71891	0.225347	FALSE

**Table 15:** Hyperparameter recommendation/ranking.

Group	Hyperparameters	Optimizer	Max Mean Accuracy	Recommendation
A	0.01, 32.0	SGDM	99.65	✓
B	0.001, 10.0	SGDM	99.13	×

**Table 16:** Comparison of classification accuracy of the presented model and other SOTA models.

Author	Year	Dataset	Method	Accuracy
Arshia Rehman et al. [46]	2020	figshare	VGG16 CNN	98.67%
Naeem ullah et al. [47]	2022	Kaggle	Inception ResNet V2 CNN	98.91%
Zahoor et al. [17]	2024	Kaggle	ResBR-Net	98.22%
Ayesha Younis et al. [16]	2024	Kaggle	ResNet-50	98.32%
Our Proposed	2024	Kaggle	ResNet-18	99.65%

**Table 17:** Self-assessment of the study’s compliance with the STARD (equator-network) checklist.

STARD Checklist Item	Description	Current Study’s Compliance
Study limitations	Include sources of potential bias, statistical uncertainty, and generalizability.	Discussed dataset homogeneity and computational setup limitations. To enhance reliability, ANOVA and Tukey HSD tests were conducted to evaluate variability and establish confidence intervals, confirming the robustness of ResNet18’s performance. These additions align with the STARD 2015 checklist, improving clarity and transparency.
Implications for practice	Address the clinical role and intended use of the index test.	The study highlights the role of ResNet18 in improving diagnosis and generalizability across diverse datasets. A detailed comparison with other state-of-the-art (SOTA) models, presented in Table 16, addresses the implications for practice as outlined in the STARD 2015 checklist. This comparison demonstrates ResNet18’s superior performance, reinforcing its potential for practical integration into clinical workflows.
Test results	Provide cross-tabulation of index test results against the reference standard.	Confusion matrices are presented in Figure 9 and Figure 10, showcasing classification accuracy on source 1 and source 2 datasets.
Estimates of diagnostic accuracy	Report estimates and their precision, such as sensitivity, specificity, and confidence intervals.	Validation metrics (accuracy, precision, recall, and F1-score) were provided for all models in Table 5 and Table 6. To enhance reliability, ANOVA and Tukey HSD tests were conducted to evaluate variability and establish confidence intervals, confirming the robustness of ResNet18’s performance. These additions align with the STARD 2015 checklist, improving clarity and transparency.
Flow of participants	Use a diagram to show the flow of participants through the study.	Participant selection and dataset descriptions are mentioned, and also framework illustration of the study is presented in Figure 3, which includes dataset preparation, training, validation, and testing stages, addressing the STARD 2015 flow of participant requirements.
Analysis methods	Specify methods for estimating or comparing diagnostic accuracy measures.	Comparative analysis is shown in Table 5 and Table 6, as well as figures for classification performance.
Scientific background	Provide clinical background and the test’s intended use.	The clinical relevance of brain tumor classification and ResNet18’s advantages are discussed.

## Data Availability

On request, the corresponding author will provide datasets that are used to categorize brain tumors.
